# Therapeutic Notes

**Published:** 1903-07

**Authors:** Nelson W. Wilson

**Affiliations:** Buffalo, N.Y.


### Therapeutic Notes.

CONDUCTED BY NELSON W. WILSON, M. D., Buffalo, N.Y.

### BUTTERMILK, A FOOD FOR INFANTS.

BAGINSKY, in the *British Medical Journal* of September 6, 1902, says he was at first reluctant to test the value of buttermilk for infants, as recommended by Dutch physicians, and therefore began sparingly in July, 1901, to use it in cases which failed to improve upon sterilised or modified milk; as improvement followed, he used it freely for other infants. From his experience he concludes that buttermilk is a good food for acutely and chronically sick infants. It is well borne after attacks of acute dyspepsia and summer diarrhea. In chronic diarrhea and chronic enteritis it may be looked upon as a life-saving preparation. He has never seen any disturbances of nutrition. The buttermilk used was made from pure cream, which was soured by means of bacteria producing lactic acid fermentation. The method of preparing the buttermilk previous to its use is fully described.

---

### CARDIAC OR RENAL DROPSY.

SHOEMAKER considers the following an excellent mixture in the dropsy due to cardiac or renal disease:

| | | | |
|---|---|---|---|
| ℞ | Bitartrate of potash | 15.0 | ℥ss. |
| | Fl. ext. convallaria | 6.0 | ʒiss. |
| | Simple syrup to make | 120.0 | ℥iv. |

---

### CONVULSIONS OF OBSCURE ORIGIN.

IN THE *Bulletin General de Therapeutique*, Perier gives the following routine in the treatment of convulsions in children: (1) loosen the clothing, so that the neck, thorax and abdomen are exposed, and lay the child on its back. If there is constipation, a rectal injection of oil, glycerin or soap should be given. (2) If there is indigestion, provoke vomiting by tickling the uvula and then give a purgative injection. (3) At the same time make the patient inhale a few drops of ether or chloroform from a handkerchief and open the windows. (4) If the convulsions are prolonged, a hot bath or mustard bath should be given; the patient should then be dried quickly; in order to prevent a return of the convulsions, one teaspoonful of the following solution should be given every hour:

| | | | |
|---|---|---|---|
| ℞ | Potassium bromid | .50 | gr. vii. ss. |
| | Sodium bromid | .50 | gr. vii. ss. |
| | Ammonium bromid | .50 | gr. vii. ss. |
| | Syrup of codeine | 5.00 | gtt. lxxv. |
| | Syrup of orange flowers | 32.00 | ʒi. |
| | Lime juice | 96.00 | ℥iii. |

In the event of the child being unable to swallow, use this injection by rectum:

| | | | |
|---|---|---|---|
| ℞ | Musk | .20 | gr. iii. |
| | Chloral hydrate | .30 | gr. iv.ss. |
| | Camphor | 1.00 | gr. xv. |
| | Yolk of egg | 10.00 | ʒii.ss. |
| | Distilled water | 96.00 | ℥iii. |

or a suppository as follows:

| | | | |
|---|---|---|---|
| ℞ | Chloral hydrate or hypnal........ | .20 to 66 | gr. iii. to x. |
| | Cacao butter .................... | 2.00 | gr. xxx. |

(5) The child should not be left alone until the convulsion is ended, which is indicated by the voiding of urine. Then search for the cause.

---

### TREATMENT OF WHOOPING COUGH.

MONTI gives a very good review of the treatment of whooping cough in the *Bulletin General de Therapeutique*, which he divides into general and local. Under the first heading, he advises that the patient be placed in a well lighted and well aired room, the temperature of which should not go below 64° and not above 68° F. If possible, the child should be taken to a temperate climate during winter. Temperature variations should be avoided in any event. Locally, inhalations of boric acid and sodium salicylate are recommended, although Monti personally prefers inhalations of carbolic acid and menthol with quinine internally. The following formula of Birch-Hirschfield's is used by Monti:

| | | | |
|---|---|---|---|
| ℞ | Carbolic acid........................ | 2.00 | gtt. xxx. |
| | Pure menthol........................ | 1.00 | gr. xv. |
| | Distilled water...................... | 192.00 | ℥vi. |

24.00 (drams vi.) of this solution should be inhaled each day.

In the internal treatment belladonna is prescribed thus:

| | | | |
|---|---|---|---|
| ℞ | Powdered belladonna .................. | .01 | gr. i.ss. |
| | Sodium bicarbonate .................. | 1.50 | gr. xxii. |
| | White sugar.......................... | 1.50 | gr. xxii. |

To be taken 1 to 3 times each day in a capsule.

Monti has found quinine to give the best results in his experience, which is large. His formula follows:

| | | | |
|---|---|---|---|
| ℞ | Quinin tannate......................... | 1.00 | gr. xv. |
| | Sodium bicarbonate..................... | 1.00 | gr. xv. |
| | White sugar ........................... | 1.00 | gr. xv. |

Divide into six (6) powders.

Dose: One every two hours.

---

### HYPODERMICS FOR HERPES ZOSTER.

SCHARFF, in *Le Progres Medical*, advocates hypodermic injections in the treatment of herpes zoster. He uses this formula:

| | | | |
|---|---|---|---|
| ℞ | Cocaine hydrochlorate........ | 0.12 to 0.24 | gr. ii. to iv. |
| | Morphine hydrochlorate ...... | 0.03 | gr. ss. |
| | Salt.......................... | 0.24 | gr. iv. |
| | Distilled water to make ...... | 120.00 | ℥iv. |

Injections should be made in the intercostal spaces near the exits of the nerves.

### LIEBREICH'S SCABIES OINTMENT.

THERE are few preparations which are so universally successful as Liebreich's ointment in the treatment of scabies. It is as follows:

| | | | |
|---|---|---|---|
| ℞ | Liquor stryax | 15.0 | ℥ss. |
| | Olive oil | 30.0 | ℥i. |

Directions: Rub well in, night and morning.

### HOW PROPERLY TO MAKE TOAST.

IN that very valuable work, "How to Cook for the Sick," are these directions for making toast, a bit of sick room cookery which is too often disregarded, and in the majority of cases improperly done. How many times has the physician found chunks of boggy and sodden, discolored bread, which has been manipulated over a flame; or pieces of fresh bread, which had been burned in an effort to make it crisp? Here is how Sachse directs toast to be made, and it cannot be improved upon. It should be given to the patient's cook—especially if there is a gas stove in the house:

The object of making toast is to convert the starch into dextrin, giving the starch its first step toward digestion and the agreeable toast flavor. The bread must first be thoroughly dried, or it cannot be brought to 400° F., the dextrinising temperature. Toast is therefore crisp to the center and a golden brown. It can be easily broken, is quickly moistened by the saliva, and, what is an advantage with invalids and children, it requires mastication.

